# Glial interleukin-1β upregulates neuronal sodium channel 1.7 in trigeminal ganglion contributing to temporomandibular joint inflammatory hypernociception in rats

**DOI:** 10.1186/s12974-018-1154-0

**Published:** 2018-04-20

**Authors:** Peng Zhang, Rui-Yun Bi, Ye-Hua Gan

**Affiliations:** 1 Central Laboratory, Peking University School and Hospital of Stomatology, 22 Zhongguancun Avenue South, Haidian District, Beijing, 100081 China; 2 Department of Oral & Maxillofacial Surgery, Peking University School and Hospital of Stomatology, 22 Zhongguancun Avenue South, Haidian District, Beijing, 100081 China; 3 Center for TMD & Orofacial Pain, Peking University School and Hospital of Stomatology, 22 Zhongguancun Avenue South, Haidian District, Beijing, 100081 China; 4 The Third Dental Center, Peking University School and Hospital of Stomatology, 10 Huayuan Lu, Haidian District, Beijing, 100088 China

**Keywords:** IL-1β, Nav1.7, Trigeminal ganglion, Satellite glial cells, Neuron, TMJ, Inflammatory hypernociception, CREB, COX-2

## Abstract

**Background:**

The proinflammatory cytokine interleukin-1β (IL-1β) drives pain by inducing the expression of inflammatory mediators; however, its ability to regulate sodium channel 1.7 (Nav1.7), a key driver of temporomandibular joint (TMJ) hypernociception, remains unknown. IL-1β induces cyclooxygenase-2 (COX-2) and prostaglandin E2 (PGE2). We previously showed that PGE2 upregulated trigeminal ganglionic Nav1.7 expression. Satellite glial cells (SGCs) involve in inflammatory pain through glial cytokines. Therefore, we explored here in the trigeminal ganglion (TG) whether IL-1β upregulated Nav1.7 expression and whether the IL-1β located in the SGCs upregulated Nav1.7 expression in the neurons contributing to TMJ inflammatory hypernociception.

**Methods:**

We treated rat TG explants with IL-1β with or without inhibitors, including NS398 for COX-2, PF-04418948 for EP2, and H89 and PKI-(6-22)-amide for protein kinase A (PKA), or with adenylate cyclase agonist forskolin, and used real-time PCR, Western blot, and immunohistofluorescence to determine the expressions or locations of Nav1.7, COX-2, cAMP response element-binding protein (CREB) phosphorylation, and IL-1β. We used chromatin immunoprecipitation to examine CREB binding to the Nav1.7 promoter. Finally, we microinjected IL-1β into the TGs or injected complete Freund’s adjuvant into TMJs with or without previous microinjection of fluorocitrate, an inhibitor of SGCs activation, into the TGs, and evaluated nociception and gene expressions. Differences between groups were examined by one-way analysis of variance (ANOVA) or independent samples *t* test.

**Results:**

IL-1β upregulated Nav1.7 mRNA and protein expressions in the TG explants, whereas NS398, PF-04418948, H89, or PKI-(6-22)-amide could all block this upregulation, and forskolin could also upregulate Nav1.7 mRNA and protein expressions. IL-1β enhanced CREB binding to the Nav1.7 promoter. Microinjection of IL-1β into the TGs or TMJ inflammation both induced hypernociception of TMJ region and correspondingly upregulated COX-2, phospho-CREB, and Nav1.7 expressions in the TGs. Moreover, microinjection of fluorocitrate into the TGs completely blocked TMJ inflammation-induced activation of SGCs and the upregulation of IL-1β and COX-2 in the SGCs, and phospho-CREB and Nav1.7 in the neurons and alleviated inflammation-induced TMJ hypernociception.

**Conclusions:**

Glial IL-1β upregulated neuronal Nav1.7 expression via the crosstalk between signaling pathways of the glial IL-1β/COX-2/PGE2 and the neuronal EP2/PKA/CREB/Nav1.7 in TG contributing to TMJ inflammatory hypernociception.

## Background

The tetrodotoxin-sensitive (TTX-S) voltage-gated sodium channel 1.7 (Nav1.7), whose principal α-subunit is encoded by the sodium channel voltage-gated type IX alpha subunit (*SCN9A*) gene, is highly expressed in the trigeminal ganglia (TG), dorsal root ganglia (DRG), sympathetic ganglia, and peripheral terminals of pain-sensing nociceptors [1, 2]. Nav1.7 amplifies weak stimuli in neurons and acts as a threshold channel for firing action potentials [3, 4]. Mutations in the SCN9A gene which lead to a gain of Nav1.7 function are associated with primary erythromelalgia [5] and paroxysmal extreme pain disorder [6], while mutations in the SCN9A gene which lead to a loss of Nav1.7 function are associated with congenital insensitivity to pain [7].

Nav1.7 plays an important role in inflammatory pain. In addition to increasing the TTX-S current (mainly include Nav1.3 and Nav1.7), Nav1.7 mRNA and protein expressions are upregulated in the DRG in a hindpaw inflammation model in rats [8]. Moreover, the role of Nav1.7 in inflammatory hypernociception is supported by knockout and knockdown studies in mice. Nociceptor-specific Nav1.7 knockout abrogates inflammation-induced mechanical and thermal hyperalgesia [9], while Nav1.7 knockdown in primary afferent neurons prevents inflammation-induced hyperalgesia [10]. We have shown that TG Nav1.7 is involved in temporomandibular joint (TMJ) inflammatory hypernociception [11]. However, Nav1.7 regulation remains poorly understood. While two studies have shown that Nav1.7 is regulated in the DRG by nerve growth factor (NGF) [12] and tumor necrosis factor-α (TNF-α) [13], the mechanisms remain to be elucidated.

Proinflammatory cytokines might be important Nav1.7 regulators. Many proinflammatory mediators, including interleukin-1 and interleukin-6 (IL-1 and IL-6), TNF-α, NGF, serotonin, and prostaglandins are increased after tissue inflammation [14]. Interleukin-1β (IL-1β), a member of IL-1 family, as the first discovered cytokine [15] is associated with peripheral sensitization [16] and the development and maintenance of inflammatory pain [17, 18]. IL-1β injection produces mechanical hypernociception in rats [19]. IL-1 receptor antagonist (IL-1ra) administration significantly reduces IL-1β-induced enhancement of nociceptive neuron responses [20] and inflammatory hyperalgesia [21]. IL-1β can increase nociceptor excitability by relieving resting slow inactivation of tetrodotoxin-resistant (TTX-R) voltage-gated sodium channels, enhancing the persistent TTX-R current near threshold levels, and increasing the TTX-S sodium current amplitude [16]. IL-1β-induced hyperalgesia is prevented by anti-NGF antibodies [17]. We hypothesized that IL-1β might modulate Nav1.7 expression to contribute to inflammatory pain.

IL-1β contributes to inflammatory pain relating to the cyclooxygenase-2 (COX-2) or prostaglandin E2 (PGE2) signaling pathway. In inflammatory pain, IL-1β has been identified as the key mediator that induces COX-2 in cultured DRG [22, 23] and TG cells [24]. IL-1β decreases mechanical nociceptive thresholds in a prostaglandin-dependent manner [25]. PGE2 is one of the major metabolites of arachidonic acid produced through the COX-2 pathway [26], and it contributes to inflammatory pain [27]. PGE2 sensitizes peripheral nociceptors through E prostanoid (EP) receptors (EP1 to EP4) [28]. Among them, the spinal EP2 receptor plays a dominant role in inflammatory pain generation [29]. PGE2 can increase TTX-S sodium channel current, including Nav1.7, in DRG neurons [30, 31]. We previously showed that PGE2 upregulates trigeminal ganglionic Nav1.7 expression through EP2 in a model of TMJ inflammatory hypernociception [32]. Therefore, our aim was to determine if IL-1β might modulate Nav1.7 expression through the COX-2/PGE2/EP2 signaling pathway to contribute to inflammatory hypernociception.

Since activation of EP2 by PGE2 stimulates adenylate cyclase to increase cAMP, which mediates events through protein kinase A (PKA) [33], and PKA activates transcription factors such as cAMP response element binding protein (CREB) [34, 35], we aimed to further determine if IL-1β might modulate Nav1.7 through the EP2-evoked PKA/CREB signaling pathway.

Cytokines from glial cells play important roles in glial-neuron communication, which contributes to pain hypersensitivity after inflammation or nerve injury [36, 37]. After inflammation or nerve injury, chemical mediators (chemokines and neurotransmitters) released from nerve terminals activate glial cells, which release a variety of mediators (such as cytokines, including IL-1β) that in turn affect neuronal activity [37, 38]. TMJ inflammation activates glial cells in both the TG and spinal trigeminal nucleus [39]. In particular, satellite glial cell (SGC) activation in the TG modulates neuronal excitability via IL-1β post-inflammation [40]. Therefore, we also examined whether IL-1β was derived from the SGCs to modulate Nav1.7 expression in neurons.

In this study, we showed that IL-1β derived from the SGCs upregulated neuronal Nav1.7 expression in the TG through the COX-2/PGE2/EP2-evoked PKA/CREB signaling pathway contributing to TMJ inflammatory hypernociception in rats.

## Methods

### Animals

Adult male and female Sprague-Dawley (SD) rats (220–250 g, Vitalriver Experimental Animal Technique Company, Beijing, China) were housed under a 12-h light/dark cycle in a pathogen-free area with ad libitum access to water and food. The experimental protocols were approved by the Animal Use and Care Committee of Peking University, China, and were consistent with the Ethical Guidelines of the International Association for the Study of Pain. All efforts were made to minimize the number of animals used and their suffering. All data were acquired from male rats, except that the data presented in Fig. 5 were acquired from both male and female rats.

### TG explant culture

Immediately after rats were deeply anesthetized with 1% pentobarbital sodium (50 mg/kg, intraperitoneal (i.p.)) and decapitated, TG were dissected and rinsed with D-Hank’s balanced buffered saline. The TGs were then incubated in 2 mL of Dulbecco’s modified eagle’s medium (DMEM) containing 1 g/L D-Glucose, L-Glutamine, and Pyruvate (Gibco, Invitrogen, USA) containing 10% heat-inactivated fetal bovine serum (FBS) in a humid incubator at 37 °C with 5% CO_2_ and 95% air for 20 min. To avoid contamination, a mixture of 100 U/mL penicillin and 100 μg/mL streptomycin was added to the media prior to the incubation [41]. The TG cultures (at least three TGs per group and tested separately) were then treated with IL-1β, NS-398, PF-04418948, H89, PKI-(6-22)-amide, or forskolin for 24 h. When drug combinations were necessary, NS-398, PF-04418948, H89, or PKI-(6-22)-amide were applied to the cultures 30 min before the application of IL-1β or forskolin. All drugs were purchased from Sigma, USA. IL-1β (I2393); NS-398 (N194); PF-04418948 (PZ0213); H89 (B1427); PKI-(6-22)-amide (P6062); forskolin (F3917).

### Real-time quantitative polymerase chain reaction (qPCR)

The TG dissected from rats were homogenized (Tissue Lyser II, QIAGEN, Germany) in TRIzol (Invitrogen, USA). Total RNA was isolated according to the manufacturer’s instructions. Reverse transcription and real-time qPCR were performed as described in detail previously [42]. The primers for rat β-actin, Nav1.7, COX-2, and IL-1β were custom-synthesized (Sangon Biotech Company, Shanghai, China) according to sequences from previous reports [32, 42]. The mRNA level of the target gene was determined from the value of the threshold cycle (Ct) relative to that of β-actin using the formula 2^-△△Ct^ (△Ct = gene of interest Ct = *β*-actin Ct, △△Ct = experimental group, △Ct = control group △Ct).

### Western blot analysis

The dissection and homogenization of the TG and Western blot analysis were performed as described in detail previously [32]. The primary antibodies were as follows: anti-Nav1.7 antibody (1:1000, 20257-1-AP, Proteintech, USA), anti-COX-2 antibody (1:1000, 12282s, Cell Signaling Technology, USA), anti-phospho-CREB antibody (1:1000, 9198s, Cell Signaling Technology, USA), anti-CREB antibody (1:1000, 9191s, Cell Signaling Technology, USA), anti-IL-1β antibody (1:500, YR0913021, R&D systems, USA), anti-glial fibrillary acidic protein (GFAP; a marker of glial activation) antibody (1:1000, P14136, Cell Signaling Technology, USA), and anti-β-actin antibody (1:10000, TA-09, ZSGB-BIO, China). The densities of the bands were quantified using the NIH ImageJ 1.38 software (NIH, Bethesda, MD, USA) and expressed as fold change of the control group after normalization to β-actin or CREB.

### Chromatin immunoprecipitation assay

A chromatin immunoprecipitation (ChIP) assay was performed as described in a previous study [43]. Briefly, TG explants were cultured at 37 °C with 5% CO_2_ and 95% air for 24 h with or without IL-1β. Approximately 1 g of TG was cross-linked with 1% formaldehyde. The samples were homogenized on ice using a mechanical homogenizer until no tissue pieces were visible. The chromatin was sonicated into fragments ranging between 500 and 1000 bp in size using a Sonifer II 450 (Branson, Danbury, CT) with a 3-mm tip set at duty cycle 20 and output level 2 and was then pulled down by anti-CREB or anti-IgG antibodies for polymerase chain reaction (PCR) amplification. The primers used to amplify the fragments containing the cAMP response element (CRE) consensus sequence (TGACG) in the rat Nav1.7 promoter were as follows: primer 1: 5′-AAA TAG CAT CCC TCT TAG-3′ (sense) and 5′-ACA GAT GAG GCC ATG CCC-3′ (anti-sense); primer 2: 5′-CGA AAG CAG CTC TAG TAA C-3′ (sense) and 5′-GGT GGT CAA GTT TGC TG-3′ (anti-sense). Anti-IgG antibody (3900, Cell Signaling Technology, USA) served as the negative control in the assay. The densities of the bands were quantified using the NIH ImageJ 1.38 software (NIH, Bethesda, MD, USA) and expressed as a fold change of the control group after normalization to input group’s band.

### Induction of TMJ inflammation

The rats were anesthetized with 1% pentobarbital sodium (40 mg/kg, i.p.). The peritemporomandibular joint tissue were injected with 50 μl complete Freund’s adjuvant (CFA; F5881, Sigma, USA; 1:1 oil:saline emulsion) or sterile saline to induce TMJ inflammation for 24 h, as described previously [42, 44, 45].

### Intratrigeminal ganglionic injections

Microinjections of reagents into the TG were performed as previously described [11, 46], with minor modifications. Our group previously performed this experiment with a high success rate [47]. Briefly, rats were deeply anesthetized with 1% pentobarbital sodium (50 mg/kg, i.p.) A small amount of 1% pentobarbital sodium (0.1–0.2 ml) subcutaneously injected if the rats were not completely anesthetized. Then, the rats mounted onto a stereotaxic frame (model 68001, RWD Life Science Company, Shenzhen, China). Two guide cannulae (10 mm in length, with 0.56 mm outer diameter and 0.32 mm inner diameter) were implanted into the rat bilateral trigeminal ganglia (3.5 mm posterior to the bregma, 3.6 mm lateral from the midline, and 9 mm ventral from the surface of the skull) through a small hole drilled in the skull. The guide cannulae were then anchored to the skull with stainless steel screws and dental self-curing acrylic resin. A stainless steel stylet was inserted into the cannula to prevent obstruction and infection. After surgery, the animals were maintained under similar preoperative conditions and fed ad libitum for at least 7 days. The head withdrawal threshold (at least five rats per group) was measured before and 1 week after the cannula implantation to examine whether the implantation affected nociception. Microinjections of 10 μl of vehicle (saline), 10 μl (10 μg) of IL-1β (Sigma, USA), or 10 μl (10 μg) of fluorocitrate (Sigma, F9634, USA) were performed into the TG with a 10 μl Hamilton microsyringe (RWD Life Science Co., Ltd., Shenzhen, China). This microsyringe was connected by a PE-10 polyethylene catheter with an inner cannula (12 mm in length, with 0.3 mm outer diameter and 0.14 mm inner diameter), which was extended by 2 mm beyond the end of the guide cannula. The injections were performed over 1 min to allow the reagents to sufficiently diffuse into the TG. The head withdrawal threshold was also measured 2 h before and 24 h after the microinjection. The rats were then microinjected with 1 μl of Evans blue into the TG using the same microinjection procedure to assess the accuracy of the injection site in the TG and decapitated. The injection locations in the TG were evaluated by dissection.

### Head withdrawal threshold measurement

The head withdrawal threshold was measured as an indicator of hypernociception following TMJ inflammation as reported previously [42, 44]. Before the head withdrawal tests, the rats were habituated to stand on their hind paws and lean against the experiment’s hand wearing a regular leather working glove. During the whole test sessions, the rats were unrestrained but remained motionless. To avoid bias, behavioral testing was performed by a researcher blinded to the experimental conditions. Each group contains at least five rats. An electronic von Frey filament (IITC Life Science, Woodland Hills, CA, USA) was applied to the rat’s TMJ with gradual and increasing forces until its head withdrew. The pressure at which the animal head was withdrawn was recorded. Head withdrawal threshold was calculated based on at least five measurements at intervals of 1 min per joint and are displayed as the mean ± standard deviation (SD).

### Immunohistofluorescence

The rats were anesthetized with an overdose of pentobarbital sodium and euthanized by transcardiac perfusion (250 mL body temperature 0.1 M phosphate buffered saline (PBS) pH 7.4, followed by 200–300 mL ice-cold 4% paraformaldehyde in 0.1 M PBS pH 7.4). After perfusion, the TG were postfixed in 4% paraformaldehyde for 4–6 h, incubated in 30% sucrose solution (in 0.1 M PBS) overnight at 4 °C and sectioned (5 μm thick) on a cryostat. The sections were then mounted on poly-l-lysine-coated slides and used for immunohistofluorescence. Immunohistofluorescence was performed as described previously [11, 42]. To avoid bias, the immunohistofluorescence was performed in a blinded manner, and the confocal microscopic images were also acquired and analyzed in a blinded manner. In addition, to minimize immunolabeling variations, the sections from all groups were processed very similarly in the same batch. For immunofluorescence, rabbit anti-Nav1.7 antibody (1:300, 20257-1-AP, Proteintech, USA), rabbit anti-COX-2 antibody (1:100, ab52237, Abcam, USA), rabbit anti-phospho-CREB antibody (1:300, 9198s, Cell Signaling Technology, USA), and goat anti-IL-1β antibody (1:100, YR0913021, R&D systems, USA) with or without mouse anti-GFAP antibody (1:300, P14136, Cell Signaling Technology, USA) were used as primary antibodies. The cells displaying immunoreactivity for GFAP, IL-1β, COX-2, phospho-CREB, Nav1.7, both GFAP and IL-1β, or both GFAP and COX-2 were counted in three randomly selected fields per section per TG (total three TGs). Only the clearly labeled cells that presented a discernable nucleus or cytoplasm were included. The number of positive cells was presented as the mean ± SD. The co-localized SGCs were calculated as the percentage of the GFAP-positive SGCs expressing of IL-1β or COX-2. And the data were presented as the mean ± SD.

### Statistical analysis

Statistical analyses were performed using SPSS 20.0 for Windows (SPSS Inc., Chicago, IL, USA). All data are presented as mean ± SD. Differences between all groups were examined by one-way analysis of variance (ANOVA) followed by Tukey’s multiple comparisons test, and the differences between two groups were examined using an independent samples *t* test. Differences with *P* < 0.05 were deemed to be statistically significant.

## Results

### IL-1β upregulated Nav1.7 expression in TG explants

**Fig. 1:**
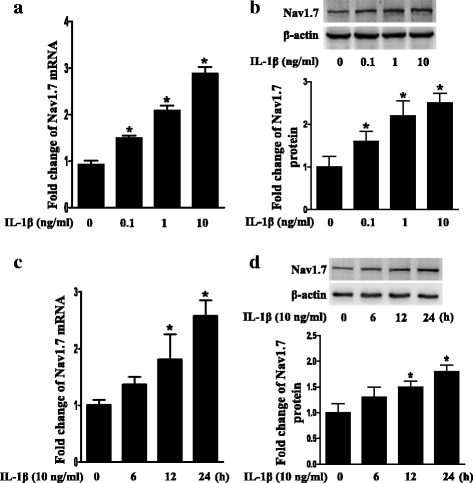
Upregulation of Nav1.7 expression in TG explants after treatment with IL-1β. **a** Dose-course of Nav1.7 mRNA expression in TG after treatment with IL-1β (0.1 to 10 ng/mL) for 24 h. **b** Dose-course of Nav1.7 protein expression in TG after treatment with IL-1β (0.1 to 10 ng/mL) for 24 h. **c** Time-course of Nav1.7 mRNA expression in TG after treatment with IL-1β (10 ng/mL). **d** Time-course of Nav1.7 protein expression in TG after treatment with IL-1β (10 ng/mL). Quantification of Nav1.7 protein expression was presented as fold change of the control group (lower panel). One-way ANOVA, *P < 0.05 versus control group; *n* = 3

We treated TG explants with IL-1β to test whether IL-1β could upregulate Nav1.7 expression. Both the mRNA and protein expression of Nav1.7 were dose and time dependently u-regulated by treatment with IL-1β (*P* < 0.05; Fig. 1a–d).

### IL-1β upregulated Nav1.7 expression depending on COX-2/PGE2/EP2 in TG explants

**Fig. 2:**
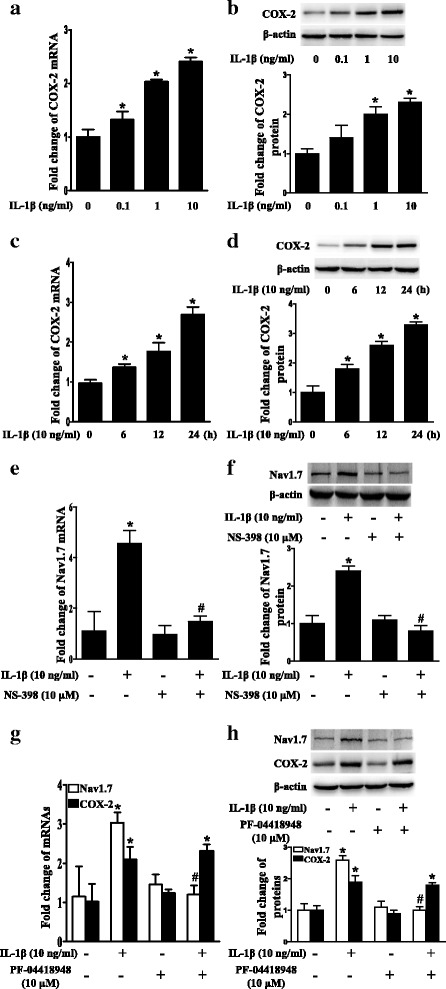
Upregulation of Nav1.7 expression by IL-1β was dependent on COX-2/PGE2/EP2 in TG explants. **a** Dose-course of COX-2 mRNA expression in TG after treatment with IL-1β (0.1 to 10 ng/mL) for 24 h. **b** Dose-course of COX-2 protein expression in TG after treatment with IL-1β (0.1 to 10 ng/mL) for 24 h. **c** Time-course of COX-2 mRNA expression in TG after treatment with IL-1β (10 ng/mL). **d** Time-course of COX-2 protein expression in TG after treatment with IL-1β. (E) IL-1β-induced upregulation of Nav1.7 mRNA expression in TG was blocked by COX-2 selective inhibitor NS398. **f** IL-1β-induced upregulation of Nav1.7 protein expression in TG was blocked by COX-2 selective inhibitor NS398. **g** EP2 selective antagonist PF-04418948 blocked upregulation of Nav1.7 mRNA expression, but not COX-2 mRNA expression in TG after treatment with IL-1β. **h** EP2 selective antagonist PF-04418948 blocked upregulation of Nav1.7 protein expression, but not COX-2 protein expression in TG after treatment with IL-1β. Quantification of protein expressions were presented as fold change of the control group (lower panel). One-way ANOVA, *P < 0.05 versus the control group, ^#^*P* < 0.05 versus IL-1β group; *n* = 3. NS398: COX-2 selective inhibitor; PF-04418948: EP2 selective antagonist

Since IL-1β mediates inflammatory pain through COX-2/PGE2 [18, 24, 48] and we previously showed that PGE2 upregulated trigeminal ganglionic Nav1.7 through EP2 [32], we examined COX-2 expression in TG explants after treatment with IL-1β. As expected, IL-1β upregulated COX-2 mRNA (Fig. 2a, c) and protein (Fig. 2b, d) expression in a dose- and time-dependent manner (*P* < 0.05). Furthermore, treatment with NS398, a COX-2 selective inhibitor, completely blocked the IL-1β-induced upregulation of Nav1.7 mRNA and protein expression, as shown in Fig. 2e, f (*P* < 0.05). Treatment with PF-04418948, an EP2 selective antagonist, also completely blocked the IL-1β-induced upregulation of Nav1.7 expression, but not COX-2 expression, as shown in Fig. 2g, h (*P* < 0.05).

### IL-1β upregulated Nav1.7 expression through the EP2-evoked PKA/CREB signaling pathway in TG explants

**Fig. 3:**
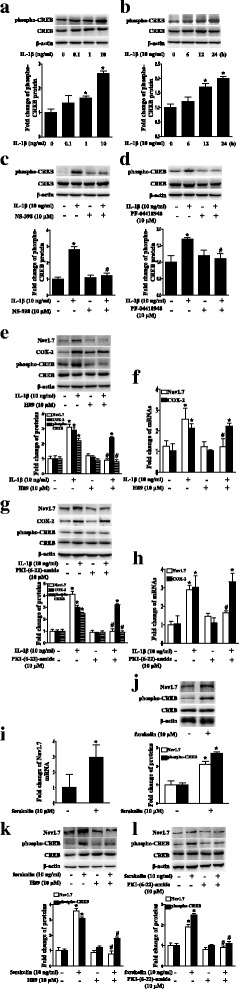
IL-1β upregulated Nav1.7 expression through the EP2-evoked PKA/CREB signaling pathway in TG explants. **a** Dose-course of phospho-CREB protein expression in TG after treatment with IL-1β (0.1 to 10 ng/mL) for 24 h. **b** Time-course of phospho-CREB protein expression in TG after treatment with IL-1β. **c** Upregulation of phospho-CREB protein expression by IL-1β in TG was blocked by COX-2 selective inhibitor NS398. **d** Upregulation of phospho-CREB protein expression by IL-1β in TG was blocked by EP2 selective antagonist PF-04418948. **e** PKA inhibitor H89 blocked IL-1β-induced upregulation of Nav1.7 and phospho-CREB protein expressions, but not COX-2 protein expression in TG. **f** PKA inhibitor H89 blocked IL-1β-induced upregulation of Nav1.7 mRNA expression, but not COX-2 mRNA expression in TG. **g** PKA inhibitor PKI-(6-22)-amide blocked IL-1β-induced upregulation of Nav1.7 and phospho-CREB protein expression, but not COX-2 protein expression in TG. **h** PKA inhibitor PKI-(6-22)-amide blocked IL-1β-induced upregulation of Nav1.7 mRNA expression, but not COX-2 mRNA expression in TG. **i** Forskolin upregulated Nav1.7 mRNA expression in TG for 24 h. **j** Adenylate cyclase agonist forskolin upregulated Nav1.7 and phospho-CREB protein expressions in TG for 24 h. **k** PKA inhibitor H89 blocked forskolin-induced upregulation of Nav1.7 and phospho-CREB protein expressions in TG. **l** PKA inhibitor PKI-(6-22)-amide blocked forskolin-induced upregulation of Nav1.7 and phospho-CREB protein expressions in TG. Quantification of protein expressions were presented as fold change of the control group (lower panel). One-way ANOVA or Independent samples *t* test, *P < 0.05 versus the control group, ^#^*P* < 0.05 versus IL-1β group; *n* = 3. NS398: COX-2 selective inhibitor; PF-04418948: EP2 selective antagonist; H89 and PKI-(6-22)-amide: inhibitors of PKA; Forskolin: adenylate cyclase agonist

Since activation of EP2 by PGE2 stimulates adenylate cyclase to increase cAMP, which mediates events through PKA [33] and PKA activates the phosphorylation of transcription factors such as CREB [34, 35], we confirmed that IL-1β upregulated phospho-CREB in a dose- and time-dependent manner (*P* < 0.05; Fig. 3a, b). We also showed that the COX-2 inhibitor NS-398 or the EP2 inhibitor PF-04418948 blocked the upregulation of phospho-CREB (*P* < 0.05; Fig. 3c, d). We then examined whether the IL-1β-induced upregulation of Nav1.7 was dependent on PKA. Since there is no selective inhibitor for PKA [49], two PKA inhibitors, H89 or PKI-(6-22)-amide, were used. The two inhibitors both completely blocked the IL-1β-induced upregulation of Nav1.7 expression and CREB phosphorylation, but not COX-2 expression (*P* < 0.05; Fig. 3e–h). Moreover, forskolin, an adenylate cyclase agonist, also upregulated Nav1.7 mRNA and protein expression in the TG (*P* < 0.05; Fig. 3i, j). In addition, H89 or PKI-(6-22)-amide blocked the forskolin-induced upregulation of Nav1.7 expression and CREB phosphorylation (*P* < 0.05; Fig. 3k, l).

### IL-1β enhanced CREB binding to the Nav1.7 promoter

**Fig. 4:**
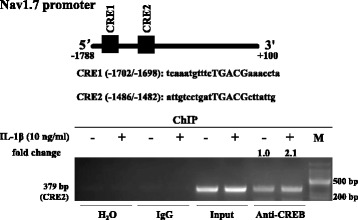
IL-1β enhanced CREB binding to the Nav1.7 promoter. The schematic diagram indicates two potential CREs (CRE1 at − 1702/− 1698 and CRE2 at − 1486/− 1482) in the rat Nav1.7 promoter (− 1788/+ 100, translation start site as + 1). ChIP assay showed that CREB could bind to CRE2, and this binding was enhanced by the treatment with IL-1β for 24 h. The densities of the bands were quantified using the NIH ImageJ 1.38 software and expressed as fold change of the control group after normalization to input band. CRE, cAMP response element

The phosphorylation of CREB activates the transcription of cAMP response element (CRE)-targeted genes. CRE consists of a palindromic sequence (TGACGTCA) [50], and many CREB binding elements consist of half-palindromic sites (TGACG) [51] or contain multiple substitutions [52, 53]. We found that there were two potential CREs in the rat Nav1.7 (SCN9A) promoter (− 1788/+ 100, translation start site as + 1) (Fig. 4). To examine whether CREB could be recruited to the Nav1.7 promoter by IL-1β, we performed a ChIP assay after treating TG explants with IL-1β for 24 h. Although there were two potential CREs (CRE1 at − 1702/− 1698 and CRE2 at − 1486/− 1482) in the rat Nav1.7 promoter, CREB only bound to CRE2 (Fig. 4), and not CRE1 (data not shown), and this binding was enhanced by treatment with IL-1β (Fig. 4).

### Intratrigeminal ganglionic injection of IL-1β resulted in decrease in the head withdrawal threshold and upregulation of Nav1.7, COX-2, and phospho-CREB expressions in both male and female rats

**Fig. 5:**
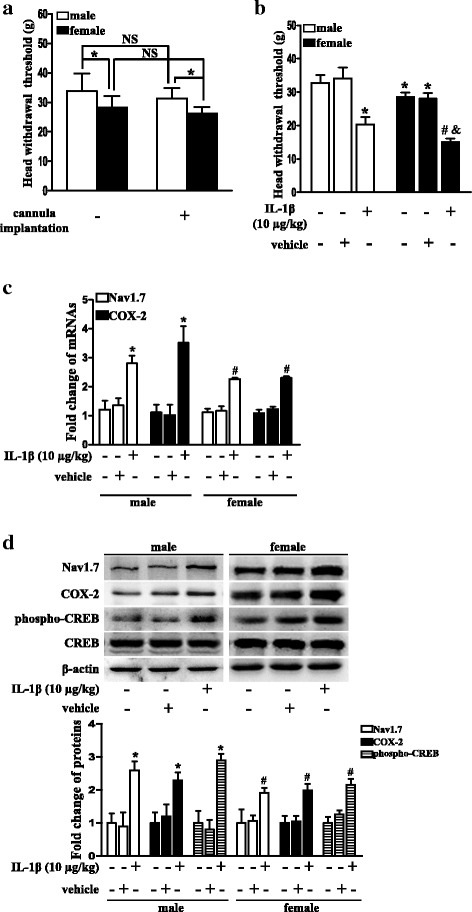
Intratrigeminal ganglionic injection of IL-1β resulted in decrease in the head withdrawal threshold and upregulation of Nav1.7, COX-2, and phospho-CREB expressions in both male and female rats. **a** The head withdrawal threshold in the TMJ region 1 week before or after cannula implantation in male and female rats. Independent samples *t* test, *P < 0.05; *n* = 15. NS, no significance. **b** The head withdrawal threshold 24 h after intratrigeminal ganglionic injection of IL-1β or vehicle in both male and female rats. One-way ANOVA, *P < 0.05 versus the male control or vehicle group, ^#^*P* < 0.05 versus the control or vehicle group of both female and male rats, ^&^*P* < 0.05 versus the male IL-1β group; *n* = 5. **c** mRNA expressions of Nav1.7 and COX-2 24 h after intratrigeminal ganglionic injection of IL-1β in male and female rats. One-way ANOVA, *P < 0.05 versus the male control or vehicle group, ^#^*P* < 0.05 versus the female control or vehicle group; *n* = 3. **d** Protein expressions of Nav1.7, COX-2, and phospho-CREB 24 h after intratrigeminal ganglionic injection of IL-1β in male and female rats. Quantification of protein expressions were presented as fold change of the control group (low panel). One-way ANOVA, *P < 0.05 versus the male control or vehicle group, ^#^*P* < 0.05 versus the female control or vehicle group; *n* = 3

We further examined the effects of IL-1β on Nav1.7 expression in vivo by direct injection of IL-1β into the TGs in both male and female rats, since gender may play a role in pain behavior [54, 55]. As shown in Fig. 5, the cannula implantation did not affect the head withdrawal threshold 1 week after implantation in both male and female rats (*P* > 0.05; Fig. 5a). However, the female rats showed lower head withdrawal threshold in the TMJ region than that in the male rats both before and after the cannula implantation (*P* < 0.05; Fig. 5a). Twenty-four hours after intratrigeminal ganglionic injection of IL-1β, the head withdrawal threshold in the TMJ region significantly decreased in both male and female rats (*P* < 0.05; Fig. 5b). However, the degree of decrease in the head withdrawal threshold in the female rats (13.01 ± 0.96; *n* = 5) did not statistically differ from that in the male rats (13.40 ± 0.55; *n* = 5) after intratrigeminal ganglionic injection of IL-1β (*P* > 0.05), although the head withdrawal threshold in the female rats was still lower than that in the male rats after intratrigeminal ganglionic injection of IL-1β (*P* < 0.05; Fig. 5b). Correspondingly, the mRNA expressions of COX-2 and Nav1.7 and the protein expressions of COX-2, phospho-CREB, and Nav1.7 in the TGs were significantly upregulated 24 h after intratrigeminal ganglionic injection of IL-1β, as compared with that of the control or vehicle group in both male and female rats (*P* < 0.05; Fig. 5c, d). However, the fold change of mRNA expression of Nav1.7 (female vs male: 1.123 ± 0.16 vs 1.503 ± 0.25; *P* > 0.05; *n* = 3) and COX-2 (female vs male: 1.144 ± 0.14 vs 2.155 ± 0.92; *P* > 0.05; *n* = 3) and the fold change of protein expression of Nav1.7 (female vs male: 0.91 ± 0.27 vs 1.68 ± 0.07; *P* > 0.05; *n* = 3), COX-2 (female vs male: 0.94 ± 0.05 vs 1.21 ± 0.10; *P* > 0.05; *n* = 3), and phospho-CREB (female vs male: 1.03 ± 0.54 vs 1.93 ± 0.11; *P* > 0.05; *n* = 3) in female rats did not statistically differ from that in the male rats after intratrigeminal ganglionic injection of IL-1β (Fig. 5c, d).

### Trigeminal ganglionic IL-1β, COX-2, phospho-CREB, and Nav1.7 were concurrently increased with hypernociception after the induction of TMJ inflammation

**Fig. 6:**
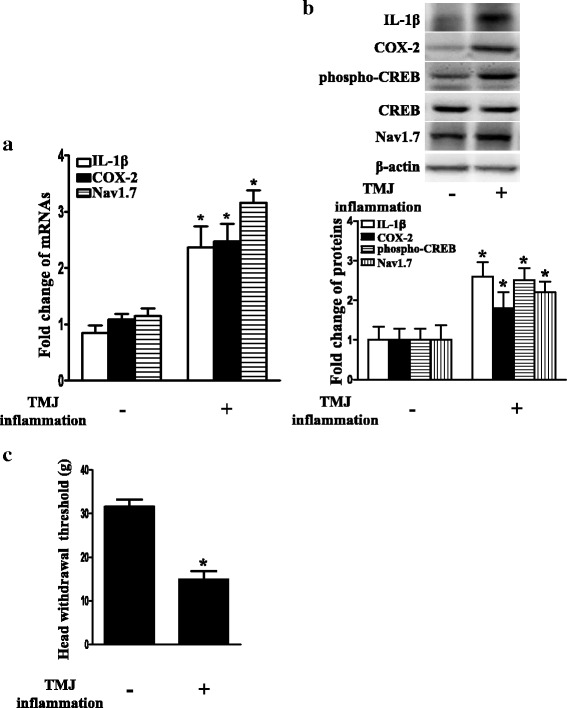
Upregulation of trigeminal ganglionic IL-1β, COX-2, phospho-CREB, and Nav1.7 accompanied with decrease in the head withdrawal threshold after induction of TMJ inflammation. **a** Upregulation of IL-1β, COX-2, and Nav1.7 mRNA expressions in TG after induction of TMJ inflammation for 24 h. Independent samples *t* test, *P < 0.05 versus the control group; *n* = 3. **b** Upregulation of IL-1β, COX-2, phospho-CREB, and Nav1.7 protein expressions in TG after induction of TMJ inflammation for 24 h. Quantification of protein expressions were presented as fold change of the control group (lower panel). Independent samples *t* test, *P < 0.05 versus the control group; *n* = 3. **c** Decrease in the head withdrawal threshold after induction of TMJ inflammation for 24 h. Independent samples *t* test, *P < 0.05 versus the control group; *n* = 5

We previously showed that the trigeminal ganglionic Nav1.7, COX-2, and PGE2 levels were increased after the induction of TMJ inflammation [32]. However, it remains to be tested whether the TMJ inflammation-induced upregulation of Nav1.7 is dependent on the IL-1β/COX-2/PGE2/EP2-evoked PKA/CREB signaling pathway in the TG. Therefore, we first examined whether TMJ inflammation could concurrently upregulate trigeminal ganglionic IL-1β, COX-2, phospho-CREB, and Nav1.7 expressions. As shown in Fig. 6a, b, the mRNA and protein expressions of these molecules were significantly upregulated in the TG after the induction of TMJ inflammation for 24 h, compared with the control group (*P* < 0.05). Conversely, the head withdrawal threshold significantly decreased after the induction of TMJ inflammation for 24 h (*P* < 0.05; Fig. 6c), suggesting that TMJ inflammation-induced hypernociception.

### TMJ inflammation-induced activation of SGCs contributed to inflammatory hypernociception through communication between glial IL-1β/COX-2 and neuronal phospho-CREB/Nav1.7

**Fig. 7:**
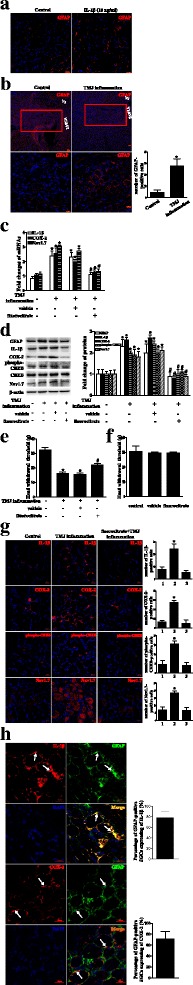
TMJ inflammation-induced SGCs activation involved in inflammatory hypernociception through communication between glial IL-1β/COX-2 and neuronal phospho-CREB/Nav1.7. **a** Confocal images of immunofluorescent staining of GFAP, which was not affected in TG explants after treatment with IL-1β for 24 h. **b** Confocal images of immunofluorescent staining of GFAP, which was increased, specifically surrounding neurons-innervating TMJ (red box), in the TG after TMJ inflammation. The number of GFAP-positive cells was presented with histogram (right panel). V3 represents the mandibular division, and V1 and V2 represent the ophthalmic and maxillary divisions. **c** TMJ inflammation-induced upregulation of IL-1β, COX-2, and Nav1.7 mRNA expressions in TG were blocked by intratrigeminal injection of SGC activation inhibitor fluorocitrate. One-way ANOVA, *P < 0.05 versus the control group; *n* = 3. **d** TMJ inflammation-induced upregulation of GFAP, IL-1β, COX-2, phospho-CREB, and Nav1.7 protein expressions in TG were blocked by intratrigeminal injection of SGC activation inhibitor fluorocitrate. Quantification of protein expressions were presented as fold change of the control group (right panel). One-way ANOVA, *P < 0.05 versus the control group; *n* = 3. **e** SGC activation inhibitor fluorocitrate partly blocked TMJ inflammation-induced decrease in the head withdrawal threshold. One-way ANOVA, *P < 0.05 versus the control group; *n* = 5. **f** The head withdrawal threshold showed no difference among the control, vehicle, and fluorocitrate groups after injection of vehicle or fluorocitrate for 24 h. One-way ANOVA, *P* > 0.05 versus the control group; *n* = 5. **g** Confocal images of immunofluorescent staining of IL-1β, COX-2, phospho-CREB, and Nav1.7 in TG. TMJ inflammation-induced increase in immunofluorescent staining of IL-1β and COX-2 in SGCs, and phospho-CREB and Nav1.7 in neurons were blocked by intratrigeminal injection of fluorocitrate. The number of IL-1β-, COX-2-, phospho-CREB-, or Nav1.7-positive cells was presented with histogram (right panel). 1: control group; 2: TMJ inflammation group; 3: fluorocitrate + TMJ inflammation group. **h** Confocal images of immunofluorescent staining of IL-1β and COX-2 co-localized with GFAP in TG. IL-1β (red) or COX-2 (red) co-stained with GFAP (green) in SGCs became orange-yellow after TMJ inflammation (as indicated by the arrows). The percentage of GFAP-positive SGCs expressing of IL-1β or COX-2 were presented with histogram (right panel). Red bar represents 20 μm. Fluorocitrate: an inhibitor of SGC activation

TMJ inflammation activates glial cells in the TG contributing to inflammatory pain [39, 40]. Increased expression of glial fibrillary acidic protein (GFAP) in SGCs around sensory neurons is a useful marker of glial activation, although the role of this molecule is still unknown [56, 57]. Although GFAP immunofluorescent staining was not affected in the TG explants after in vitro treatment with IL-1β for 24 h (Fig. 7a), GFAP immunofluorescent staining was profoundly stronger in SGCs surrounding neurons-innervating TMJ in the TG after TMJ inflammation for 24 h when compared with the control group (*P* < 0.05; Fig. 7b). To examine whether the TMJ inflammation-induced upregulation of neuronal Nav1.7 expression in the TG and inflammatory hypernociception were dependent on the activation of SGCs in the TG, we injected fluorocitrate, an inhibitor of SGC activation, into the TG before the induction of TMJ inflammation. Fluorocitrate has been used in previous studies [58, 59] that showed that the direct injection of fluorocitrate into the TG or DRG causes a significant reduction in the activation of SGCs. As shown in Fig. 7c–e, fluorocitrate completely blocked the TMJ inflammation-induced upregulation of GFAP, IL-1β, COX-2, phospho-CREB, and Nav1.7 expressions and partially blocked TMJ inflammation-induced hypernociception (*P* < 0.05). No difference in nociception was observed after intratrigeminal ganglionic injection of fluorocitrate or vehicle for 24 h (*P* > 0.05; Fig. 7f). Immunohistofluorescence further confirmed that the TMJ inflammation-induced increase in IL-1β and COX-2, which co-stained with GFAP respectively, which was located in the SGCs, whereas phospho-CREB and Nav1.7 were found in neurons in the TG, and that the increases were all blocked by fluorocitrate (*P* < 0.05; Fig. 7g, h).

## Discussion

In this study, we showed that glial IL-1β could upregulate neuronal Nav1.7 expression in the TG contributing to TMJ inflammatory hypernociception. First, IL-1β upregulated Nav1.7 mRNA and protein expression in TG explants. This upregulation of Nav1.7 expression was blocked by the COX-2 selective inhibitor or selective antagonist for PGE2 receptor EP2. Second, the upregulation of Nav1.7 by IL-1β was also blocked by PKA inhibitors or induced by forskolin, an adenylate cyclase agonist. Third, IL-1β upregulated phospho-CREB and enhanced the binding of CREB to the Nav1.7 promoter. Fourth, intratrigeminal ganglionic injection of IL-1β also induced the upregulation of trigeminal ganglionic Nav1.7 expression and this corresponded with hypernociception in the TMJ region. Fifth, induction of TMJ inflammation activated the SGCs in TG and induced IL-1β and COX-2 expressions in the SGCs, and phospho-CREB and Nav1.7 expressions in the neurons. Intratrigeminal ganglionic injection of an inhibitor of SGCs activation completely blocked the TMJ inflammation-induced SGCs activation and upregulation of these genes and also alleviated TMJ inflammation-induced hypernociception. To the best of our knowledge, this is the first report to demonstrate that glial IL-1β upregulated neuronal Nav1.7 expression in the TG contributing to TMJ inflammatory hypernociception. These results may help us to further understand the nociceptive effects of IL-1β in inflammatory pain, the regulation of Nav1.7 expression, and the communication between glia and neurons. They may also aid in the development of a new strategy to deal with inflammatory pain.

IL-1β was an important regulator of Nav1.7. Although IL-1β alters primary neural activity or sensitivity and contributes to inflammatory pain [16, 19, 60], the exact target of IL-1β remains to be identified. Here, we showed that IL-1β upregulated Nav1.7 expression in TG both in vitro and in vivo and that injection of IL-1β into the TG could induce TMJ hypernociception. Considering that the biophysical property of Nav1.7 is to amplify weak stimuli and to act as a threshold channel for firing action potentials in neurons [4] and that an upregulation of Nav1.7 expression accompanies the increase in TTX-S current amplitude in neurons [8], injection of IL-1β into the TGs induced hypernociception of TMJ region at least partially via upregulating trigeminal ganglionic Nav1.7 expression. Our finding of an upregulation of Nav1.7 induced by IL-1β could be a new mechanism for IL-1β contributing to inflammatory pain. Given that Nav1.7 is a unique pain gene in which mutations that result in loss-of-function leads to a congenital inability to experience pain [7], upregulation of Nav1.7 by IL-1β is also of theoretical and practical significance. Understanding the targets of IL-1β and the regulators of Nav1.7 may help in the development of new anti-inflammatory pain drugs. Although gender may play a role in pain behavior [54, 55] and the female rats showed lower head withdrawal thresholds than the male rats before and after cannula implantation and after intratrigeminal ganglionic injection of IL-1β in our study, intratrigeminal ganglionic injection of IL-1β did not induce more hypernociception in the female rats than in the male rats, since the degree of decrease in the head withdrawal threshold in the female rats did not statistically differ from that in the male rats after intratrigeminal ganglionic injection of IL-1β. In addition, the molecular events, i.e., the fold change of COX-2, phospho-CREB, and Nav1.7 expressions, also did not support that there was a difference between the male and female after intratrigeminal ganglionic injection of IL-1β. The reason why the female rats showed lower head withdrawal threshold than the male rats in our study is still unknown. Considering that the inclusion of the female rats in our study was too preliminary due to the neglecting of estrous cycles, whether the trigeminal ganglionic Nav1.7 expression was involved in the gender difference of responses to mechanical stimuli remains to be determined by future studies.

IL-1β upregulated Nav1.7 expression through the COX-2/PGE2/EP2-evoked PKA/CREB signaling pathway. While IL-1β contributes to inflammatory pain depending on COX-2/PGE2 [23, 61], and the EP2-activated PKA/CREB signaling pathway plays an important role in inflammatory pain [62, 63], whether Nav1.7 is regulated by the COX-2/PGE2/EP2-activated PKA/CREB signaling pathway remains to be determined. Here, we first showed that the upregulation of Nav1.7 expression by IL-1β was dependent on the COX-2/PGE2/EP2 signaling pathway. The key evidence included the facts that IL-1β upregulated COX-2 expression and the COX-2 selective inhibitor NS398 and EP2 selective antagonist PF-04418948 both blocked the effects of IL-1β on Nav1.7 expression, strongly indicating that IL-1β-induced Nav1.7 was dependent on COX-2 and EP2. Then, we proved that the EP2-evoked PKA/CREB signaling pathway mediated the IL-1β-induced upregulation of trigeminal ganglionic Nav1.7 expression as follows: IL-1β upregulated phospho-CREB and Nav1.7 expressions in the TG, whereas the PKA inhibitors, H89 or PKI-(6-22)-amide, blocked the effects of IL-1β on phospho-CREB and Nav1.7, while the adenylate cyclase agonist forskolin mimicked the effect of IL-1β on phospho-CREB and Nav1.7. Using a ChIP assay, we also proved that IL-1β enhanced CREB binding to the Nav1.7 promoter, therefore leading to an upregulation of Nav1.7 mRNA expression and subsequently protein expression. In addition, the IL-1β-induced phosphorylation of CREB was also blocked by the COX-2 selective inhibitor NS398 and EP2 selective antagonist PF-04418948. These results suggest that the IL-1β-induced upregulation of Nav1.7 is dependent on the EP2-activated cAMP/PKA/CREB signaling pathway. Our results strongly suggested that Nav1.7 was a target for the IL-1β-induced COX-2/PGE2 and EP2-evoked PKA/CREB signaling pathway.

Glial IL-1β upregulated neuronal Nav1.7 expression in the TG after TMJ inflammation contributing to inflammatory hypernociception. Although the communication between glial cells and neurons is important during the development of inflammatory pain [37, 39, 64], the mechanism by which this occurs is not fully understood. Here, we showed that TMJ inflammation activated trigeminal ganglionic SGCs, which was demonstrated by the increase in GFAP immunofluorescent staining. This result was consistent with those from previous studies, in which GFAP, as a marker of SGC activation, is increased in the TG after peripheral inflammation [39, 40]. Furthermore, TMJ inflammation resulted in an increase in expressions of IL-1β and COX-2 in the SGCs, as indicated by the co-localization of IL-1β or COX-2 with GFAP, and increases in phospho-CREB and Nav1.7 in the neurons in the TG. The localization of IL-1β, phospho-CREB, and Nav1.7 demonstrated in our study was well similar to previous studies [11, 40, 65]. However, the localization of COX-2 in our study was somewhat different to that shown in two previous studies [24], in which, after stimulation with IL-1β, COX-2 was observed in both TG glial cells and neurons [24] or cultured DRG neurons [66]. In contrast, in our study, COX-2 appeared to be only expressed in the SGCs in the TG after the induction of TMJ inflammation. The reasons for this difference are unknown, thought it may be related to the condition of the cells. The previous studies stained for COX-2 in cultured TG or DRG neurons after IL-1β treatment, whereas we stained for COX-2 in TG frozen sections after the induction of TMJ inflammation. In addition, we directly injected the SGCs activation inhibitor fluorocitrate into the TG and observed that this inhibitor completely blocked TMJ inflammation-induced activation of SGCs and upregulation of IL-1β, COX-2, phospho-CREB, and Nav1.7 in the TG. These results implied that the TMJ inflammation-induced upregulation of Nav1.7 expression was dependent on the activation of SGCs. SGC activation also appeared to be required for the upregulation of glial IL-1β expression following TMJ inflammation, since our in vitro assay showed that exogenous IL-1β did not activate SGCs, but it still upregulated COX-2 and Nav1.7 expressions in the TG explants for 24 h (Fig. 7a). Our results showing that exogenous IL-1β did not activate glial cells also differ from those of a previous study in which GFAP was shown to be increased after treatment of cultured TG glial cells with IL-1β [24]. The reasons for this difference are not clear, though they may also be related to the different conditions of the cells.

**Fig. 8:**
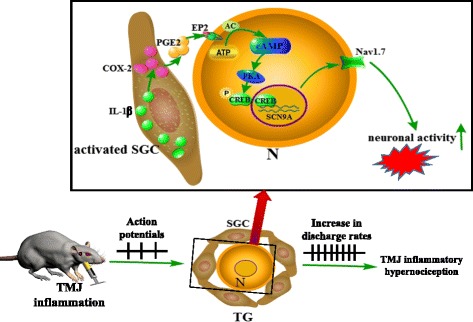
Diagram of glial IL-1β-induced upregulation of neuronal Nav1.7 contributed to TMJ inflammatory hypernociception through the COX-2/PGE2/EP2-evoked PKA/CREB signaling pathway. TMJ inflammation somehow activates SGCs, which activate the transcriptions of IL-1β and COX-2 leading to increase in PGE2 release from the SGCs to subsequently activate the neuronal EP2/PKA/CREB signaling pathway then leading to upregulation of Nav1.7 in neurons and finally the increase in the neuronal excitability, which amplifies the stimuli in the TMJ contributing to the development of TMJ inflammatory hypernociception. TG, trigeminal ganglion; N, neuron; SGC, satellite glial cells

Given that the SGC inhibitor only partially blocked TMJ inflammation-induced hypernociception but the inhibitor completely blocked the TMJ inflammation-induced upregulation of Nav1.7 expression, it seemed that the activation of SGCs might serve as an “amplifier” for nociception via activation of the glial IL-1β/COX-2/PGE2 signaling pathway to subsequently activate the neuronal EP2/PKA/CREB/Nav1.7 signaling pathway, finally leading to hypernociception in the TMJ region. A schematic diagram was shown in Fig. 8 to illustrate this potential process. Other mechanisms, such as the sensitization of neurons, Schwann cells that can produce TNF-α, and infiltrating immune cells, such as macrophages and lymphocytes, also play a role in development of inflammatory pain [36, 67, 68]. To some extent, these might explain why the intratrigeminal ganglionic injection of an SGC inhibitor only partially reverses the hypernociception of the inflamed TMJ.

Owing to a lack of commercially available antibodies derived from different species for sufficient combinational use for our immunofluorescent assays, we could not co-stain for IL-1β, COX-2, and GFAP in the same SGCs, and even co-stain for phospho-CREB and Nav1.7 in the same neurons. It was noted that the use of saline as the control for CFA in our study was not proper. Incomplete Freund’s adjuvant should have been used as the control of CFA, which only lacks the mycobacterial but other components are same to CFA, to excludes the effects of other components on Nav1.7 expression. Moreover, it still needs to be elucidated how TMJ inflammation activates SGCs and then activates the transcription of IL-1β within them in the TG. Although little is known about the signaling mechanisms linking peripheral inflammation and SGC activation, it is speculated that SGC activation is dependent on signals released by primary neurons in the TG [69]. Further studies are needed to test this.

## Conclusions

In conclusion, we have shown that glial IL-1β upregulated neuronal Nav1.7 expression in the TGs via the crosstalk between signaling pathways of the glial IL-1β/COX-2/PGE2 and the neuronal EP2/PKA/CREB/Nav1.7, contributing to TMJ inflammatory hypernociception. Our results may aid in the understanding of TMJ inflammatory pain and the development of a new strategy to deal with TMJ inflammatory pain.

## Abbreviations

CFA: Complete Freund’s adjuvant
COX-2: Cyclooxygenase-2
CRE: cAMP response element
CREB: cAMP response element binding protein
DRG: Dorsal root ganglia
GFAP: Glial fibrillary acidic protein
IL-1ra: IL-1 receptor antagonist
IL-1β: Interleukin-1β
Nav1.7: Sodium channel 1.7
NGF: Nerve growth factor
PGE2: Prostaglandin E2
PKA: Protein kinase A
*SCN9A*: Sodium channel voltage-gated type IX alpha subunit
SGCs: Satellite glial cells
TG: trigeminal ganglion
TMJ: Temporomandibular joint
TNF-α: Tumor necrosis factor-α
TTX-R: Tetrodotoxin-resistant
TTX-S: Tetrodotoxin-sensitive
